# Genetic Variation of 25 Y-Chromosomal and 15 Autosomal STR Loci in the Han Chinese Population of Liaoning Province, Northeast China

**DOI:** 10.1371/journal.pone.0160415

**Published:** 2016-08-02

**Authors:** Jun Yao, Bao-jie Wang

**Affiliations:** School of Forensic Medicine, China Medical University, Shenyang, 110122, China; Harvard Medical School, UNITED STATES

## Abstract

In the present study, we investigated the genetic characteristics of 25 Y-chromosomal and 15 autosomal short tandem repeat (STR) loci in 305 unrelated Han Chinese male individuals from Liaoning Province using AmpFISTR^®^ Yfiler^®^ Plus and Identifiler^TM^ PCR amplification kits. Population comparison was performed between Liaoning Han population and different ethnic groups to better understand the genetic background of the Liaoning Han population. For Y-STR loci, the overall haplotype diversity was 0.9997 and the discrimination capacity was 0.9607. Gene diversity values ranged from 0.4525 (DYS391) to 0.9617 (DYS385). *Rst* and two multi-dimensional scaling plots showed that minor differences were observed when the Liaoning Han population was compared to the Jilin Han Chinese, Beijing Han Chinese, Liaoning Manchu, Liaoning Mongolian, Liaoning Xibe, Shandong Han Chinese, Jiangsu Han Chinese, Anhui Han Chinese, Guizhou Han Chinese and Liaoning Hui populations; by contrast, major differences were observed when the Shanxi Han Chinese, Yunnan Bai, Jiangxi Han Chinese, Guangdong Han Chinese, Liaoning Korean, Hunan Tujia, Guangxi Zhuang, Gansu Tibetan, Xishuangbanna Dai, South Korean, Japanese and Hunan Miao populations. For autosomal STR loci, DP ranged from 0.9621 (D2S1338) to 0.8177 (TPOX), with PE distributing from 0.7521 (D18S51) to 0.2988 (TH01). A population comparison was performed and no statistically significant differences were detected at any STR loci between Liaoning Han, China Dong, and Shaanxi Han populations. The results showed that the 25 Y-STR and 15 autosomal STR loci in the Liaoning Han population were valuable for forensic applications and human genetics, and Liaoning Han was an independent endogenous ethnicity with a unique subpopulation structure.

## Introduction

Liaoning Province, located in the northeast of China, is known in Chinese as “the Golden Triangle” from its shape and strategic location. It was established in 1907 as the name of Fengtian and changed to Liaoning in 1929, with an estimated population of approximately 43.91 million in 2014 (www.stats.gov.cn). The population is mostly Han Chinese (83.94%) with minorities of Manchus (12.88%), Mongols (1.60%), Hui (0.632%), Koreans (0.576%) and Xibe (0.317%). Liaoning Han individuals mainly migrated from Shandong Peninsula during the hundred-year period starting at the last half of the 19^th^ century. “Chuang Guandong” is a description that Han Chinese population, especially from the Shandong Peninsula and Zhili, entered Manchuria [1]. During the first two centuries of the Manchu Qing Dynasty, Liaoning Province is the traditional homeland of the ruling Manchus with only certain Manchu Bannermen, Mongol Bannermen, and Chinese Bannermen allowed in. The region, now known as Northeast China, has an overwhelmingly Han population. After the establishment of the People's Republic of China at the end of the Chinese Civil War, further immigrations were organized by the Central Government to "develop the Great Northern Wilderness", eventually peaking the population over 100 million people [2, 3]. Thus, it is necessary and sufficient to investigate the genetic background of Liaoning Han population and compare the genetic distance with other population. Additionally, it is interesting to observe how much admixture took place over the past 100 years among Han Chinese and other groups.

Y-chromosomal short tandem repeats (Y-STR) is a useful tool for inferring genetic genealogy evolution [4] and ancient human migration trajectories and timing [5, 6]. The non-recombinant region of the Y-chromosome may play a potential role in revealing the ethnic and regional representation of the Han Chinese population owing to its significant phylogeographic information content [7, 8]. It can supply an informative reference for investigating patterns of genetic variation in the Han Chinese population across East Asia considering that the genetic and cultural diversity among East Asian populations is still not fully understood. Autosomal STR loci are usually applied in forensic personal identification and paternity tests, which can provide a mighty powerful discrimination capability without influenced by linkage disequilibrium. It can also be used to uncover the population genetic backdrop and structure [9]. The population data of autosomal STR loci can be utilized to constructed the phylogenies and clarify the genetic structure using genetic distance measurements, neighbor-joining dendrograms and principal component analysis base on different genotyping frequencies [10].

Therefore, we investigated the frequencies of 25 Y-STR and 15 autosomal STR loci in Liaoning Han population to expand the available population information for forensic medicine and human genetic diversity. Population comparison was performed between Liaoning Han population and different ethnic groups to better understand the genetic background of the Liaoning Han population.

## Methods

### Study population

Three hundred and five blood samples were collected from unrelated healthy male individuals living in Liaoning Province, Northeast China, after obtaining written informed consent. The blood was then stained onto filter papers. Samples were obtained and analyzed after approval from the Ethics Committee of China Medical University.

### Data extraction, PCR amplification, and genotyping

Genomic DNA was extracted using Chelex-100 [11]. PCR amplification was performed using AmpFISTR^®^ Yfiler^®^ Plus and Identifiler^TM^ PCR amplification kits (Thermo Fisher Scientific, CA, USA) in a GeneAmp^®^ PCR 9700 (Thermo Fisher Scientific, CA, USA) thermal cycler, according to respective manufacturer specifications. The AmpFISTR^®^ Yfiler^®^ Plus amplification kit (Thermo Fisher Scientific, Waltham, MA, USA) can co-amplify 25 Y-STR loci with six dyes, including seven rapidly mutating loci [12]. The AmpFISTR^®^ Identifiler^TM^ PCR Amplification kit (Thermo Fisher Scientific) can co-amplify 15 autosomal STR loci and the Amelogenin locus with five dyes. Fragments of the 25 Y-chromosomal and 15 autosomal STR loci were produced simultaneously. Separation and detection of amplicons was performed on an Applied Biosystems™ 3500 Series Genetic Analyzer (Thermo Fisher Scientific, Waltham, MA, USA). Data were analyzed using GeneMapper *ID* v4.1 software (Thermo Fisher Scientific, Waltham, MA, USA). Control DNA 007 was included as a standard reference in each batch of genotyping. We strictly followed the recommendations of the DNA Commission of the International Society of Forensic Genetics (ISFG) for Y-STR analysis [13].

### Data analysis

For Y-STR loci, allele frequencies and gene diversity were calculated using PowerMarker v3.25 [14]. Haplotype frequencies, random match probabilities (sum of squares) and haplotype diversity were calculated using Arlequin Software v3.5 [15]. The discrimination capacity (DC) was determined as the proportion of different haplotypes in each sample [16]. A cluster structure of Y-STR haplotypes was generated using the YHRD database (http://www.yhrd.org/). To compare data from the studied Liaoning Han population with other published data, genetic distance (*Rst* statistics) was measured by analysis of molecular variance (AMOVA) and visualized using two multi-dimensional scaling (MDS) *Rst* plots via YHRD online tools (http://www.yhrd.org/Analyse/AMOVA).

For autosomal loci, sample allele frequencies and exact Hardy-Weinberg equilibrium (HWE) tests were calculated using PowerMarker v3.25 [14]. Values for power of discrimination (DP), polymorphism information content (PIC), power of exclusion (PE), and heterozygosity (He) were calculated using Power Stats v1.2 software [17] that had been modified by Raquel, et al. to support and manage the large amount of samples [18]. Pairwise genetic distance (*Fst*) and *p values* for each locus were calculated between populations using Arlequin v3.5 software [15]. Furthermore, Nei’s standard genetic distance between populations was generated by the Phylip 3.69 package [19] and visualized with Treeview software [20]. Because the published relevant data is limited, the included groups for population comparison between Y-STR and autosomal STR are different.

## Results and Discussion

### Y-chromosomal STR

**Fig 1:**
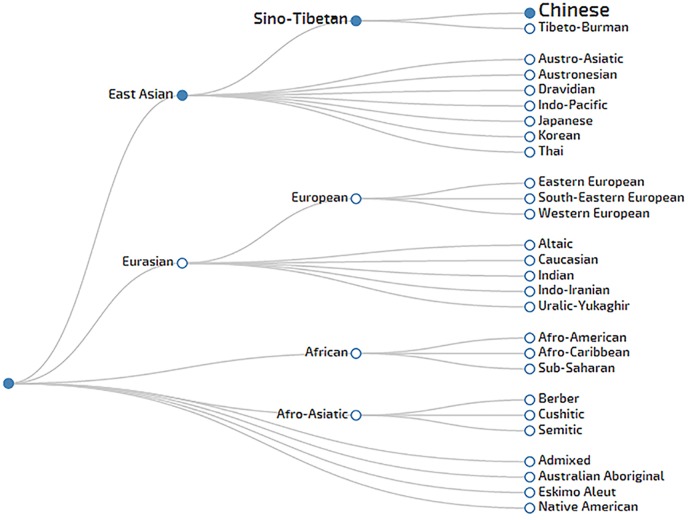
A cluster structure based on haplotypes of the Liaoning Han population in the YHRD database.

Two hundred and ninety-three different haplotypes were observed from 305 unrelated individuals. Among them, 281 were unique and 12 were shared by two individuals (S1 Table). Null alleles were found in nine individuals at DYS448 and one individual at DYS385, respectively. Haplotype diversity rendered a high value (0.9997 ± 0.0003). Likewise, a high random match probability (0.0035) was determined with a DC of 0.9607. Genetic diversity values of the 25 loci ranged from 0.4525 (DYS391) to 0.9617 (DYS385) (S2 Table). Among them, allele frequencies ranged from 0.7016 (DYS438) to 0.0033 (DYS389I, DYS389II, DYS458, YGATAH4, DYS448, DYS391, DYS456, DYS439, DYS481, DYS533, DYS576, DYS627, DYS460, DYS518, DYS449, DYF387S1 and DYS385). Cluster analysis was performed for the 12 haplotypes that were observed twice. Ancestry information showed that the haplotypes of the Liaoning Han population most likely belonged to the East Asian-Sino Tibetan-Chinese culture, which corresponds with its history, culture, and geographical distribution (Fig 1). The powerful informative content of the 25 Y-STR loci in the Liaoning Han population will be useful and interesting in forensic medicine and enrich the Han Chinese population database.

### Autosomal STR

The distribution of allele frequencies, forensic efficiencies, and statistical parameters across the 15 autosomal STR loci are presented in S3 and S4 Tables. Among the 157 observed alleles, allele frequencies ranged from 0.5164 (TH01) to 0.0016 (D8S1179, D21S11, D7S820, D3S1358, D13S317, D16S539, D2S1338, D19S433, D18S51, D5S818 and FGA). The DP ranged from 0.9621 (D2S1338) to 0.8177 (TPOX), with PE distributing from 0.7521 (D18S51) to 0.2988 (TH01). The span of He was 0.8787 (D18S51) to 0.6066 (TH01). Except for CSF1PO (0.6993), D3S1358 (0.6775), TH01 (0.5973), and TPOX (0.5872), all autosomal STR loci were highly polymorphic (PIC > 0.7) with the most D18S51 (0.8435). No departures from HWE were observed after Bonferroni’s correction for multiple testing (*p* < 0.05/15).

### Population comparison

**Fig 2:**
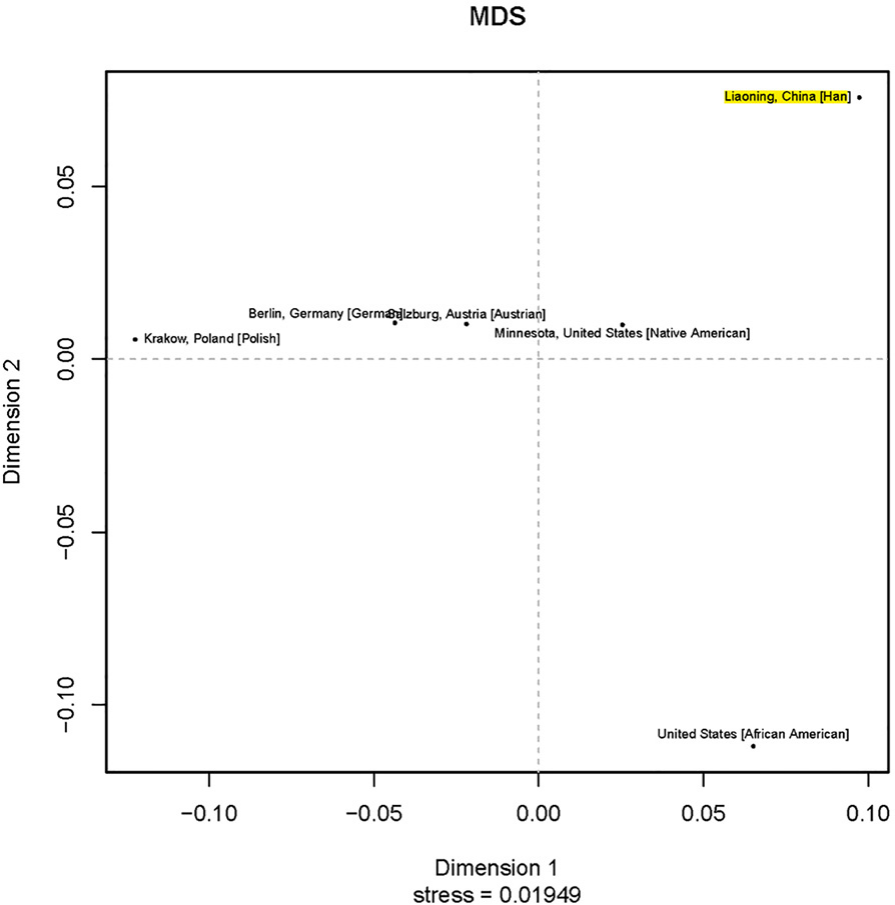
A MDS plot based on Rst between the Liaoning Han population and five reference populations (the Liaoning Han population is marked with yellow).

**Fig 3:**
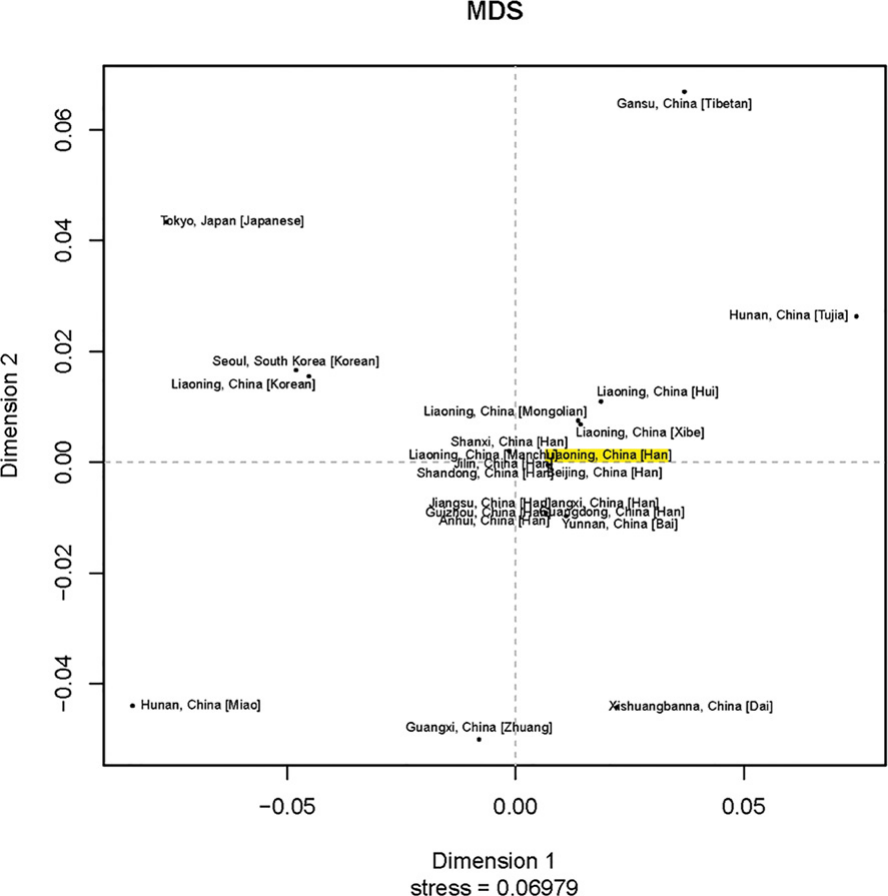
A MDS plot based on Rst between the Liaoning Han population and 22 East Asian populations (the Liaoning Han population is marked with yellow).

For Y-STR loci, we compared our haplotype data with that of the five populations that were submitted to the YHRD database (Release 51), which included Austrian [21], German [22], Polish [23], African and Native American [24]. *Rst* values for genetic distance demonstrated that haplotypes of the Liaoning Han population were significantly different from those of the other five populations (all *p* values < 0.05/5 after Bonferroni correction). As shown in the MDS plot (Fig 2), there were significant differences between Liaoning Han population and the five population. Furthermore, in order to comprehensively investigate the genetic substructure of Liaoning Han population, the population comparison using the 16 shared Y-STR loci except for DYS627, DYS460, DYS518, DYS449, DYF387S1, DYS481, DYS533, DYS576 and DYS570 was performed between Liaoning Han and 22 East Asian groups. They included Anhui Han Chinese [25], Beijing Han Chinese [26], Guangdong Han Chinese [27], Guizhou Han Chinese (YP001096), Jiangsu Han Chinese [25], Jiangxi Han Chinese [25], Jilin Han Chinese [28], Shandong Han Chinese [29], Shanxi Han Chinese [30], Yunnan Bai (YP000902), Xishuangbanna Dai (YP000903), Liaoning Hui (YP000819), Liaoning Korean [31], Liaoning Manchu [16], Hunan Miao (YP001038), Liaoning Mongolian [32], Gansu Tibetan (YP001032), Hunan Tujia (YP001037), Liaoning Xibe [33], Guangxi Zhuang (YP000591), Japanese [34] and Korean [35]. *Rst* values for genetic distance demonstrated that haplotypes of the Liaoning Han population were significantly different from those of the other 22 populations (all *p* values < 0.05/22 after Bonferroni correction; Table 1). As shown in the MDS plot (Fig 3), minor differences were observed when the Liaoning Han population was compared to the Jilin Han Chinese, Beijing Han Chinese, Liaoning Manchu, Liaoning Mongolian, Liaoning Xibe, Shandong Han Chinese, Jiangsu Han Chinese, Anhui Han Chinese, Guizhou Han Chinese and Liaoning Hui populations; by contrast, major differences were observed when the Liaoning Han population was compared to Shanxi Han Chinese, Yunnan Bai, Jiangxi Han Chinese, Guangdong Han Chinese, Liaoning Korean, Hunan Tujia, Guangxi Zhuang, Gansu Tibetan, Xishuangbanna Dai, South Korean, Japanese and Hunan Miao populations. Additionally, the populations’ distributions in the MDS plot corresponded with their respective ethno-geographic origins. It is clear that Liaoning Han Chinese has a close genetic distance with Southern Han population and Liaoning native minorities, which indicated that Liaoning Han integrated gradually with natives, such as Manchu, Mongolian and Xibe, following its geographical migration.

**Table 1:** Rst values for pairwise comparisons between populations.

Population	Liaoning, China [Han]	Anhui, China [Han]	Beijing, China [Han]	Guangdong, China [Han]	Guizhou, China [Han]	Jiangsu, China [Han]	Jiangxi, China [Han]	Jilin, China [Han]	Shandong, China [Han]	Shanxi, China [Han]	Yunnan, China [Bai]	Xishuangbanna, China [Dai]	Liaoning, China [Hui]	Liaoning, China [Korean]	Liaoning, China [Manchu]	Hunan, China [Miao]	Liaoning, China [Mongolian]	Gansu, China [Tibetan]	Hunan, China [Tujia]	Liaoning, China [Xibe]	Guangxi, China [Zhuang]	Tokyo, Japan [Japanese]	Seoul, South Korea [Korean]
Liaoning, China [Han]	-	0.0000	0.0000	0.0000	0.0000	0.0000	0.0000	0.0000	0.0000	0.0000	0.0000	0.0000	0.0000	0.0000	0.0000	0.0000	0.0000	0.0000	0.0000	0.0000	0.0000	0.0000	0.0000
Anhui, China [Han]	0.0066	-	0.0000	0.0000	0.0000	0.0000	0.0000	0.0000	0.0000	0.0000	0.0000	0.0000	0.0000	0.0000	0.0000	0.0000	0.0000	0.0000	0.0000	0.0000	0.0000	0.0000	0.0000
Beijing, China [Han]	0.0019	0.0016	-	0.0000	0.0000	0.0000	0.0000	0.0000	0.0000	0.0000	0.0000	0.0000	0.0000	0.0000	0.0000	0.0000	0.0000	0.0000	0.0000	0.0000	0.0000	0.0000	0.0000
Guangdong, China [Han]	0.0180	0.0040	0.0090	-	0.0000	0.0000	0.0000	0.0000	0.0000	0.0000	0.0000	0.0000	0.0000	0.0000	0.0000	0.0000	0.0000	0.0000	0.0000	0.0000	0.0000	0.0000	0.0000
Guizhou, China [Han]	0.0078	-0.0015	0.0050	0.0025	-	0.0000	0.0000	0.0000	0.0000	0.0000	0.0000	0.0000	0.0000	0.0000	0.0000	0.0000	0.0000	0.0000	0.0000	0.0000	0.0000	0.0000	0.0000
Jiangsu, China [Han]	0.0055	-0.0003	0.0074	0.0164	0.0057	-	0.0000	0.0000	0.0000	0.0000	0.0000	0.0000	0.0000	0.0000	0.0000	0.0000	0.0000	0.0000	0.0000	0.0000	0.0000	0.0000	0.0000
Jiangxi, China [Han]	0.0168	0.0012	0.0070	-0.0032	-0.0005	0.0156	-	0.0000	0.0000	0.0000	0.0000	0.0000	0.0000	0.0000	0.0000	0.0000	0.0000	0.0000	0.0000	0.0000	0.0000	0.0000	0.0000
Jilin, China [Han]	0.0000	0.0047	-0.0015	0.0123	0.0068	0.0103	0.0104	-	0.0000	0.0000	0.0000	0.0000	0.0000	0.0000	0.0000	0.0000	0.0000	0.0000	0.0000	0.0000	0.0000	0.0000	0.0000
Shandong, China [Han]	0.0051	0.0075	0.0008	0.0124	0.0096	0.0163	0.0079	0.0001	-	0.0000	0.0000	0.0000	0.0000	0.0000	0.0000	0.0000	0.0000	0.0000	0.0000	0.0000	0.0000	0.0000	0.0000
Shanxi, China [Han]	0.0126	0.0171	0.0092	0.0198	0.0169	0.0269	0.0126	0.0067	0.0074	-	0.0000	0.0000	0.0000	0.0000	0.0000	0.0000	0.0000	0.0000	0.0000	0.0000	0.0000	0.0000	0.0000
Yunnan, China [Bai]	0.0127	0.0022	0.0051	0.0065	0.0071	0.0129	0.0066	0.0089	0.0082	0.0143	-	0.0000	0.0000	0.0000	0.0000	0.0000	0.0000	0.0000	0.0000	0.0000	0.0000	0.0000	0.0000
Xishuangbanna, China [Dai]	0.0666	0.036	0.0419	0.0221	0.0354	0.0656	0.0277	0.0495	0.0404	0.0360	0.0219	-	0.0000	0.0000	0.0000	0.0000	0.0000	0.0000	0.0000	0.0000	0.0000	0.0000	0.0000
Liaoning, China [Hui]	0.0085	0.022	0.0190	0.0384	0.0211	0.0157	0.0406	0.0140	0.0241	0.0163	0.0321	0.0961	-	0.0000	0.0000	0.0000	0.0000	0.0000	0.0000	0.0000	0.0000	0.0000	0.0000
Liaoning, China [Korean]	0.0489	0.0564	0.0410	0.0523	0.0538	0.0714	0.0331	0.0378	0.0335	0.0371	0.0523	0.0837	0.0784	-	0.0000	0.0000	0.0000	0.0000	0.0000	0.0000	0.0000	0.0000	0.0000
Liaoning, China [Manchu]	0.0021	0.0054	-0.0003	0.0115	0.0071	0.0107	0.0084	-0.0013	0.0011	0.0074	0.0079	0.0445	0.0160	0.0365	-	0.0000	0.0000	0.0000	0.0000	0.0000	0.0000	0.0000	0.0000
Hunan, China [Miao]	0.1049	0.1127	0.1015	0.0975	0.0986	0.1315	0.0748	0.0941	0.0899	0.0591	0.1222	0.1535	0.1147	0.0669	0.0956	-	0.0000	0.0000	0.0000	0.0000	0.0000	0.0000	0.0000
Liaoning, China [Mongolian]	0.0025	0.0177	0.0129	0.0340	0.0190	0.0123	0.0294	0.0064	0.0162	0.0186	0.0277	0.0899	0.0029	0.0524	0.0104	0.0957	-	0.0000	0.0000	0.0000	0.0000	0.0000	0.0000
Gansu, China [Tibetan]	0.0639	0.0804	0.0823	0.1096	0.0887	0.0787	0.0995	0.0674	0.0874	0.0734	0.0765	0.1323	0.0463	0.1118	0.0808	0.1533	0.0481	-	0.0000	0.0000	0.0000	0.0000	0.0000
Hunan, China [Tujia]	0.0542	0.0682	0.0777	0.1088	0.0755	0.0485	0.1116	0.0696	0.0940	0.0793	0.0899	0.1839	0.0325	0.1705	0.0794	0.2044	0.0398	0.0721	-	0.0000	0.0000	0.0000	0.0000
Liaoning, China [Xibe]	0.0043	0.0155	0.0105	0.0311	0.0199	0.0123	0.0314	0.0055	0.0146	0.0149	0.0201	0.0742	0.0055	0.0635	0.0088	0.1072	0.0009	0.0485	0.0430	-	0.0000	0.0000	0.0000
Guangxi, China [Zhuang]	0.0574	0.0459	0.0552	0.0287	0.0299	0.0610	0.0270	0.0562	0.0519	0.0490	0.0520	0.0607	0.0689	0.0906	0.0536	0.0861	0.0661	0.1150	0.1452	0.0695	-	0.0000	0.0000
Tokyo, Japan [Japanese]	0.0970	0.1019	0.0932	0.0978	0.0942	0.1173	0.0822	0.0883	0.0840	0.0883	0.1041	0.1542	0.1209	0.0301	0.0856	0.1109	0.0935	0.1131	0.2085	0.1163	0.1217	-	0.0000
Seoul, South Korea [Korean]	0.0667	0.0751	0.0561	0.0701	0.0729	0.0887	0.0509	0.0553	0.0480	0.0592	0.0732	0.1039	0.1034	0.0009	0.0514	0.0937	0.0733	0.1431	0.2005	0.0883	0.1203	0.0348	-

**Fig 4:**
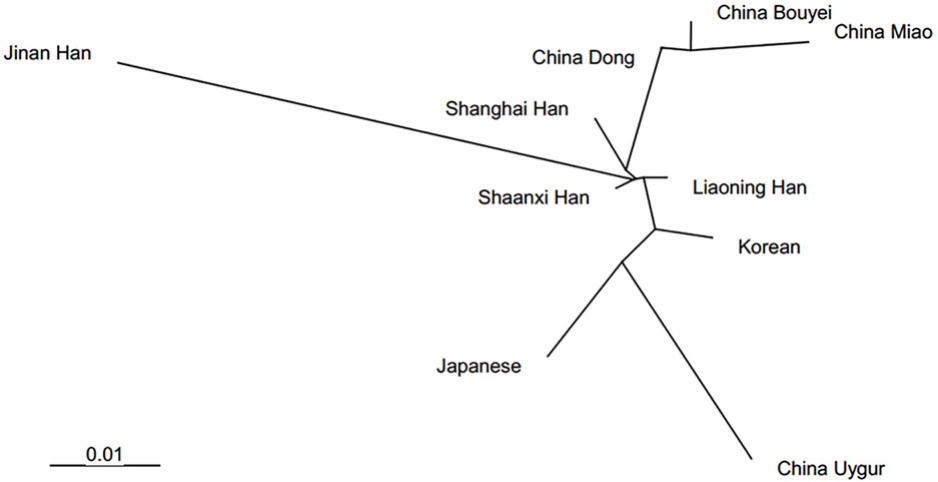
A phylogenetic tree showing the relationship between the Liaoning Han ethnic group and nine other populations. The tree was constructed using allele frequency data from 15 autosomal STR loci for all populations (Shananxi Han; Jinan Han; Shanghai Han; China Dong; China Bouyei; China Miao; China Uygur; Japanese; Korean).

For autosomal STR loci, S5 Table presents pairwise *Fst* and *p* values for differentiation tests between the Liaoning Han ethnic group and nine additional published populations [36–44]; statistically significant differences (*p* < 0.05/15) were found between the Liaoning Han population and the China Miao population at five STR loci, the China Bouyei population at four STR loci, the China Uygur and Jinan Han populations at three STR loci, the Japanese population at two STR loci, and the Korean and Shanghai Han populations at one STR locus. No statistically significant differences were detected at any STR loci between the Liaoning Han and the China Dong or Shaanxi Han populations. Table 2 shows genetic distances between populations. Fig 4 indicates clusters of unrooted phylogenetic trees to mirror the historical and geographical backgrounds of the populations compared. In culture custom, because most people in Northeast China trace their ancestries back to the migrants from the Chuang Guandong era, Northeastern Chinese were more culturally uniform compared to other geographical regions of China. Therefore, people from the Northeast would first identify themselves as "Northeasterners" before affiliating to individual provinces and cities (http://chinaneast.xinhuanet.com).

**Table 2:** Genetic distances between all pairs of populations (Phylip software according to Nei).

Population	Liaoning Han	China Bouyei	China Dong	China Miao	China Uygur	Japanese	Shaanxi Han	Shanghai Han	Jinan Han	Korean
Liaoning Han	-									
China Bouyei	0.021242	-								
China Dong	0.014437	0.006857	-							
China Miao	0.027501	0.013362	0.010337	-						
China Uygur	0.036308	0.050582	0.042861	0.063749	-					
Japanese	0.020228	0.041212	0.029945	0.042715	0.032500	-				
Shaanxi Han	0.005461	0.017733	0.013645	0.027183	0.030150	0.023933	-			
Shanghai Han	0.010897	0.018921	0.017645	0.031206	0.038085	0.028383	0.009637	-		
Jinan Han	0.050450	0.064416	0.063353	0.079116	0.074344	0.074333	0.050168	0.053725	-	
Korean	0.011539	0.030519	0.023232	0.035671	0.034461	0.017134	0.013007	0.015367	0.061562	-

For Han Chinese population, the previous studies showed that it was intricately sub-structured and clustered roughly to two (northern Han and southern Han) or three (northern Han, central Han and southern Han) subgroups [45–47]. The distinction between southern and northern Han populations were reported by Chu et al using the neighbor-joining method based on the data of STR loci [48]. The Han Chinese group has the same predecessors, the Yan Emperor and the Yellow Emperor in the Yellow River Basin. However, the Han population has been forming a series of relationships with different groups and coexisted with other ethnic groups since thousands of years ago [49]. Obviously, Liaoning Han population belonged to northern Han subgroup according to the geographic distribution and historical cultural.

The population comparison based on Y-STR loci showed that Liaoning Han was an independent endogenous ethnicity with a unique subpopulation structure. The previous study showed that Liaoning Han had a close genetic distance with Manchu, which was not as near as Han population of Jilin and Beijing, but nearer than other ethnic groups [50]. This result might indicate that the Liaoning Han integrated gradually with natives, such as Manchu, Mongolian and Xibe, following its geographical migration, which was corresponded with the historical records [9]. However, autosomal STR population comparison presented that there was no significant difference between the Liaoning Han and the China Dong or Shaanxi Han populations, which seemed to be contradictory to Y-STR results. This might be due to the discrepancy of different genetic markers. Consequently, Liaoning Han population owns its unique genetic characteristics that are different from Han population from other provinces, except for Jilin Han population.

There were two potential limitations in the present study. First, the analysis of the Y chromosomal and autosomal STR loci could not provide the precise and reliable data for population comparison with the absence of the whole genome data. Second, the included groups for population comparison between Y-STR and autosomal STR are different, due to the limited available relevant data. Thus, more genetic investigations need to do in order to better understand the characteristics of Liaoning Han Chinese population.

## Conclusion

The population comparison demonstrates that the Liaoning Han population is an independent endogenous ethnicity and still owns its unique genetic characteristics. In summary, the reported genetic characteristics of the 25 Y-STR and 15 autosomal STR loci allelic frequencies and haplotype distributions of the Liaoning Han population are informative for forensic investigation and paternity testing. The results could help inferring the genetic genealogy evolution and ancient human migration patterns.

## Supporting Information

S1 Table The haplotype distributions of the 25 Y-STR loci in the Liaoning Han population (n = 305). (XLS)

S2 Table Allele frequencies of the 25 Y-STR loci in the Liaoning Han population (n = 305). (XLS)

S3 Table Genotyping distributions of the 15 autosomal STR loci in the Liaoning Han population (n = 305). (XLS)

S4 Table The distributions of allele frequencies, forensic efficiencies, and statistical parameters of the 15 autosomal STR loci in the Liaoning Han population (n = 305). (XLSX)

S5 Table Pairwise *Fst* for differentiation tests between the Liaoning Han population (n = 305) and nine other published populations. (XLSX)

## Abbreviations

STR: short tandem repeat
AMOVA: analysis of molecular variance
MDS: multi-dimensional scaling
HWE: Hardy-Weinberg equilibrium
DP: power of discrimination
PIC: polymorphism information content
PE: power of exclusion
He: heterozygosity
Fst: Pairwise genetic distance
